# Air–Water Interfacial Adsorption of the Chaperone Protein DNAJB6b

**DOI:** 10.1021/acs.langmuir.5c01237

**Published:** 2025-07-16

**Authors:** Jon Pallbo, Marco Fornasier, Sara Linse, Ulf Olsson

**Affiliations:** † Physical Chemistry, 5193 Lund University, P.O. Box 124, Lund 221 00, Sweden; ‡ Biochemistry and Structural Biology, 5193 Lund University, P.O. Box 124, Lund 221 00, Sweden

## Abstract

Aberrant protein aggregation into amyloid fibrils is often catalyzed by interfaces. Therefore, it is important to characterize the surface activity of chaperone proteins having the ability to suppress amyloid formation. The air–water interface is of large practical significance in experimental setups used to study aggregation kinetics *in vitro*, but in addition, the binding of chaperones to hydrophobic patches on their clients may also be considered as a consequence of interfacial interactions. Here, we have studied the air–water interfacial adsorption of the human chaperone protein DNAJB6b by using hanging drop tensiometry. The dynamic surface tension exhibited a characteristic pattern in a concentration-dependent manner. First, there was an induction period during which the surface tension was close to that of the buffer and then the surface tension quite suddenly decreased, followed by a final semistable regime. DNAJB6b formed an apparently irreversibly adsorbed and elastic surface layer on the timescale of the experiments (about 2 h). The collapse of the surface layer and micelle-like clustering of DNAJB6b in the bulk likely both limit the highest attainable surface pressure. We developed a theoretical model that could successfully reproduce the main features of the results. In addition to the relevance for this specific chaperone system, the adsorption behavior of DNAJB6b was similar to that of other proteins. Thus, the framework for the model we propose might also be significant for protein adsorption in general.

## Graphical abstract

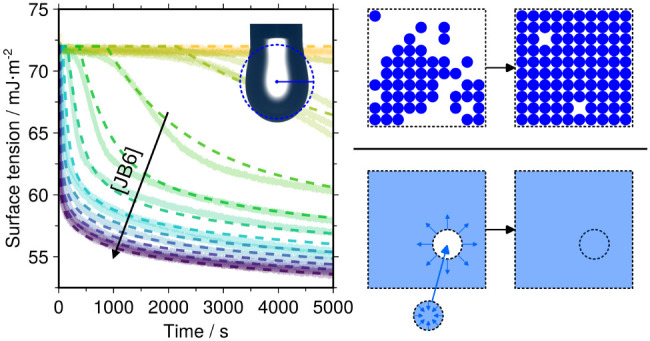

## Introduction

Molecular chaperones are a diverse set of proteins that ensure a healthy proteome by promoting proper folding and preventing misfolding and aberrant aggregation of other proteins.^1–3^ Interfaces, for example those between water and biological membranes or between water and air, are ubiquitous and often play key roles in the nucleation of amyloid fibrils, as well as for protein aggregation and denaturation in general.^4^ As just one example, in dialysis-related amyloidosis disease the surface materials used in the dialysis devices might trigger the amyloid-formation of the protein β2-microglobulin.^5^ Due to the prominence of interfaces in the formation of aberrant protein aggregates, both *in vivo* and *in vitro*, it is interesting to evaluate how chaperones behave at interfaces, since this could substantially modulate the protein aggregation kinetics. Amyloid formation is commonly studied through *in vitro* kinetic experiments, and in such setups the air–water interface may be of great practical significance. Furthermore, a recurrent part of the action of chaperone proteins is association with exposed hydrophobic patches of the client proteins, which may also be framed as an interfacial interaction. There can be commonalities in the behavior at different types of interfaces. For example, there is some evidence that the behavior of α-synuclein at the air–water interface resembles its behavior at lipid membranes in that the adsorbed protein adopts a partially α-helical structure in both cases.^6^ Thus, the study of one interface could serve as a general model for interfacial behavior, or at the very least provide key insights and serve as a reference system for comparison with other interfaces.

DNAJB6b is expressed throughout the tissues of the human body.^7,8^ It belongs to the class of proteins having an N-terminal so-called J-domain of about 70 residues forming an α-helix-rich structure. This domain enables the interaction with Hsp70 proteins, that in turn are part of a ubiquitous ATP-dependent chaperone system.^1–3^ Variants of this system are present in essentially all living organisms. Apart from the J-domain, DNAJB6b has a β-sheet rich C-terminal domain. These two domains are connected by a relatively flexible linker region. It differs from another splice variant, DNAJB6a, by being present throughout the cell, whereas the latter is localized to the nucleus.^8^ DNAJB6b (hereafter referred to as JB6) has been shown to form highly polydisperse protein aggregates at concentrations above roughly 0.1 μM under the same conditions as used in the present study (pH 8.0, moderate ionic strength).^9–11^ It is also known to be a potent ATP-independent inhibitor of amyloid formation on its own, such as for amyloid-β ^12–15^ and α-synuclein.^16–18^ The inhibition of amyloid formation can be observed even at deeply substoichiometric concentrations of JB6. Such low concentrations of the chaperone are insufficient to significantly sequester the client proteins on a one-to-one basis, suggesting that the mechanism of inhibition of amyloid formation relies on interaction with other parts of the system, such as amyloidogenic oligomers and interfaces.^12^ For this reason, having a proper model that can explain the behavior of the chaperone at interfaces is pivotal in understanding the dynamics of the chaperone-client interactions *in vitro* and *in vivo*.

In the present study, we have investigated the adsorption to the air–water interface of the human chaperone protein DNAJB6b by measuring surface tension. We measured the time and concentration dependence of the processes. We also demonstrated the apparent irreversibility of adsorption and the elasticity of the adsorbed layer, and we constructed a theoretical model that reproduces the overall behavior of the system. To the best of our knowledge, the present work is the first study characterizing the interfacial activity of a chaperone protein based on this approach. Several examples of the interfacial adsorption of proteins have been reported suggesting that this is a general phenomenon,^19–40^ with pioneering studies by Irving Langmuir.^41,42^ Yet, a consensus understanding is still lacking at the level reached for small-molecule surfactants. We therefore also hope that our work contributes to setting an explanatory framework for protein systems in a broader sense.

## Materials and Methods

### Materials

The chemicals used to prepare buffer solutions for the surface tension measurements were NaH_2_PO_4_ (Fisher BioReagents, #BP329–1), NaOH (Sigma-Aldrich, #06203), and ethylenediaminetetraacetic acid (EDTA) (Sigma-Aldrich, #E9884). Ultrapure water was provided by a Milli-Q IQ 7000 system (Merck). JB6 (241 residues, 27 × 10^3^ g/mol) was expressed and purified as previously described,^43^ except that the lyophilization between the first and second size-exclusion step was replaced by ammonium sulfate precipitation to fit the whole protocol within one working day. Aliquots of JB6 in buffer after the second size-exclusion step were flash frozen and stored at −20 °C.

### Sample Preparation

For the surface tension and QCM-D measurements, a frozen aliquot with JB6 was taken out and thawed at room temperature. The concentration of JB6 was confirmed by light absorption at 280 nm (Labbot, Probation Laboratories Sweden AB, Sweden) using an extinction coefficient of 14 400 M^–1^·cm^–1^. The solution was diluted to the desired concentrations in buffer. The buffer used in the frozen aliquots and for all samples was 20 mM Na_2_HPO_4_/NaH_2_PO_4_ + 0.2 mM EDTA at pH 8.0. The equilibration of JB6 clusters after dilution is known to be slow at room temperature^9^ but is much faster at elevated temperatures. Therefore, the diluted samples were equilibrated for 3 h at 37 °C, followed by an additional equilibration for at least 2 days at room temperature before the measurements.

### Dynamic Surface Tension Measurements

The surface tension was measured by the hanging drop method^44,45^ using a PAT1M instrument (Sinterface Technologies e.K, Germany). Measurements were performed at room temperature in an enclosed chamber, such that the rate of evaporation was low. The protocols for the measurements were automatically controlled through the instrument software. Pure water (72.0 mJ/m^2^ at 25 °C) and the buffer had the same surface tension within the measurement uncertainty and were used as reference samples. For all measurements except the ones for which the bulk was exchanged, the surface area was rapidly expanded from 6 to 50 mm^2^ (corresponding to a drop volume of about 40 μL) at the start of the measurement. For bulk exchange measurements, a coaxial capillary was used and the volume of the drops ranged from about 10–50 μL (20–55 mm^2^).

### Quartz Crystal Microbalance with Dissipation Monitoring Measurements

The adsorption of JB6 to hydrophilic surfaces was evaluate by means of quartz crystal microbalance with dissipation monitoring (QCM-D) using a Q-Sense E4 system (Biolin Scientific, Gothenburg, Sweden) with 4 measurement cells. Quartz surfaces (QSensor QSX 303 SiO_2_) with a top coating material of SiO_2_ were used (4.95 ± 0.05 MHz, 14 mm diameter, 0.3 mm thickness, and a mass sensitivity factor of 17.7 ng/cm^2^). Prior to any measurements, the crystals were cleaned by following the protocol suggested by the manufacturer: rinsing with pure water, rinsing with 99.5% ethanol, drying with N_2_ and plasma cleaning for 10 min. The crystals were introduced to the measurement cells immediately after cleaning. Pure water was then introduced to the cells via a peristaltic pump (Ismatec IPC-N 4, Zürich, Switzerland) and then left to equilibrate while monitoring the fundamental frequency (Δ*f*) and dissipation (Δ*D*) signals for about 30 min. After acquiring baselines first in water and then in buffer for at least 20 min each, the samples of JB6 at different concentrations in buffer were injected at a flow rate of 0.150 mL/min. Each sample was left to adsorb for at least 1 h before rinsing with the buffer and then with water.

The measurement data were baseline-corrected and plotted in Matlab R2024b. The adsorbed mass coupled with water was evaluated using the Sauerbrey equation.^46^

## Results and Discussion

### The Time and Concentration Dependence of the Adsorption

The surface tension of the JB6 solutions as a function of time exhibited three kinetic regimes (Figure 1a). In the first regime (1), the surface tension remained at the value of the pure buffer (approximately 72.0 mJ/m^2^). In the second regime (2), the surface tension suddenly decreased, until it reached the third regime (3), in which the surface tension value partially stabilized. The duration of the first regime, which can be referred to as an induction time, was highly concentration dependent (Figure 1b). It ranged from many minutes at the lowest concentrations decreasing to being too short to measure at the highest concentrations (above 1 μM JB6). A similar induction time in the dynamic surface tension has been reported for many proteins.^19–31,33^ For example, the induction time for hen egg-white lysozyme was almost 3 h in a Langmuir trough at a concentration of about 7 mg/L.^19^ The slope of the second regime, as well as the transition to the third regime, also depended on the bulk protein concentration. The semistable surface tension value of the third regime decreased with increasing concentration and then gradually became almost constant at JB6 concentrations above 1 μM (Figure 1c). However, the surface tension still slowly continued to decrease as a function of time for all samples during several hours. In addition to an induction time, such a slow continued surface tension decrease is characteristic of the dynamic surface tension of proteins.^20,21,32^ This distinguishes the surface tension behavior of protein solutions from those of many small-molecule surfactants, which reach stable values characterized by a thermodynamic equilibrium.^20^

**Figure 1:**
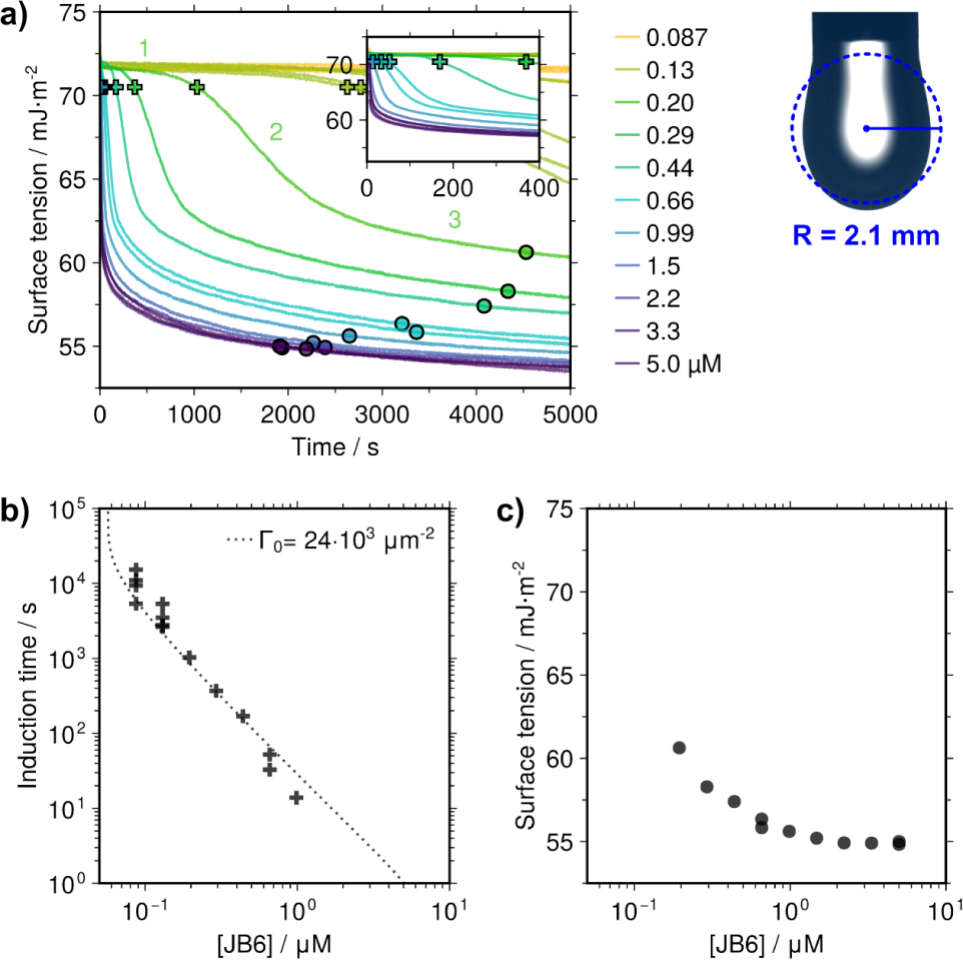
Dynamic surface tension of JB6 solutions (room temperature, 20 mM Na_2_HPO_4_/NaH_2_PO_4_ + 0.2 mM EDTA at pH 8.0). **a)** The surface tension as a function of time for solutions with different concentrations of JB6. The dynamic surface tension had three kinetic regimes, and these are indicated with numbers for the 0.20 μM JB6 curve as an example. The first (1) is an induction time during which the surface tension is close to that of the buffer. The second (2) is a rapid fall of the surface tension. The third (3) is a partial stabilization of the surface tension. The extent of these regimes changed with concentration. An example of the drop used for the measurements is also shown. For diffusion calculations, the drop was approximated as a sphere with a radius of 2.1 mm (dashed circle). The induction time, marked with crosses, was taken as the time at which the surface tension had dropped by 1.5 mJ/m^2^ below the value for the buffer. The semistable surface tension in the third kinetic regime for each concentration was taken as the value at which the rate of decrease became less than 2.5 mJ/m^2^ per hour (circles). **b)** The induction time plotted against the JB6 concentration. The dashed line is the induction time required for diffusion limited adsorption of 24 000 protein molecules per μm^2^. **c)** The semistable surface tension plotted against the JB6 concentration. The semistable surface tension gradually reached a limiting value at about 55 mJ/m^2^ with increasing JB6 bulk concentration.

The induction time has been discussed on several occasions,^21–31^ and one highly plausible explanation for this feature in the dynamic surface tension is a two-dimensional phase separation at the surface during the adsorption process (Figure 2).^47^ There is also some direct experimental evidence for this mechanism.^19^ Brewster angle microscopy in the case of JB6 also supports this picture (Section S1 and Figure S2). The protein molecules that reach the surface aggregate into a condensed phase, and not until an amount of protein sufficient to completely cover the surface with this condensed phase does the surface tension begin to decrease or, equivalently, the surface pressure begin to increase (Π = γ_0_ – γ). This is similar to a transition from a two-phase region with a constant chemical potential to a one-phase region with an increasing chemical potential.

**Figure 2:**
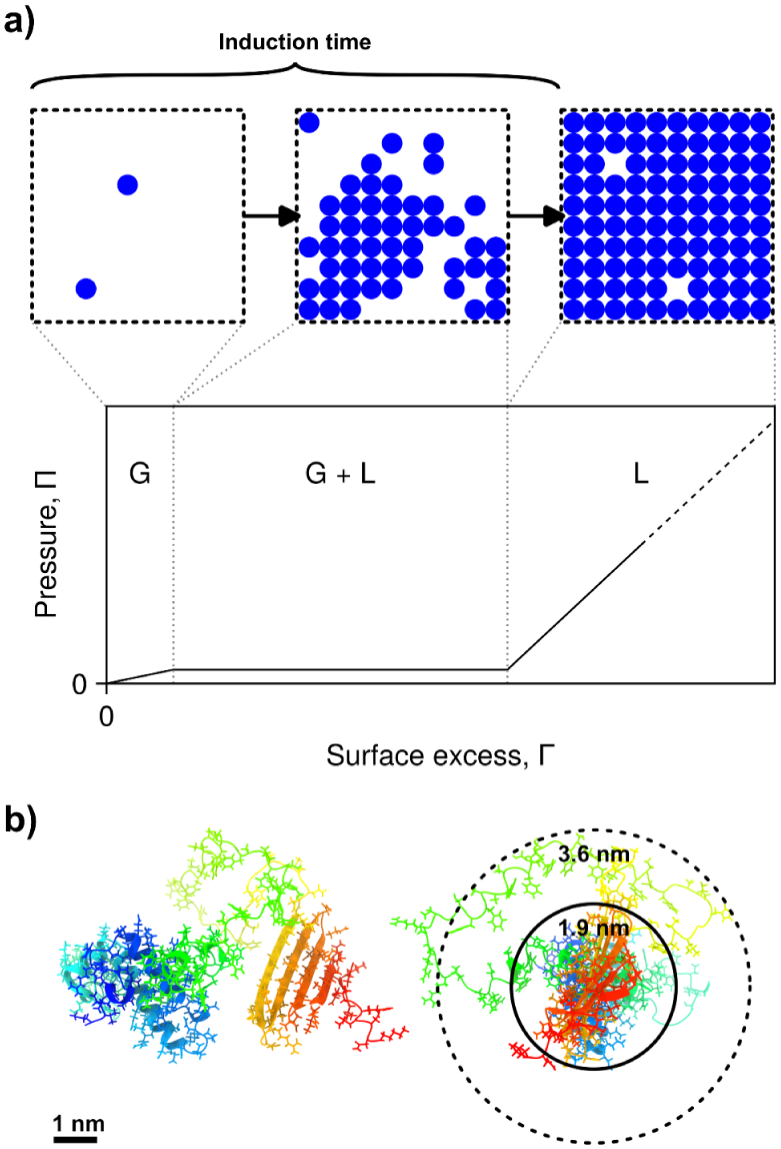
Schematic illustration of the proposed origin of the induction time, and area per molecule at the surface relative to the size of JB6. **a)** As the protein is gradually adsorbing to the air–water interface, it starts off in a two-dimensional, gas-like state (G) followed by a two-phase regime (G + L). In the two-phase regime the surface pressure, Π, and thereby the surface tension (γ = γ_0_ – Π), remains constant. Within this framework, the transition to the two-phase regime for JB6 happens when the surface pressure is still very close to zero. At the end of the induction time, there is complete surface coverage with the condensed phase (L), and adsorption of more protein leads to an increase of the surface pressure. **b)** Two perpendicular views of the structure of JB6, as predicted by AlphaFold2 ^52^ and rendered with UCSF ChimeraX.^53^ The protein has an α-helix rich J-domain at the N-terminus. This is connected to a β-sheet rich C-terminal domain by a relatively flexible linker region. The dashed circle in the left view is an estimate of the area per molecule at the end of the induction time (Γ_0_ ≈ 24 × 10^3^ μm^–2^, *r* ≈ 3.6 nm). The circle with the solid line is an estimate of the area per molecule at which the elastic surface layer starts to collapse (Γ_collapse_ ≈ 84 × 10^3^ μm^–2^, *r* ≈ 1.9 nm).

While the dynamic surface tension exhibited an induction time, this was not observed in the case of JB6 adsorption onto silica as measured in QCM-D experiments. Overall, a sharp decrease in terms of frequency was observed for all concentrations, with the highest adsorption rate at the highest JB6 concentration (0.75 μM) (Figure S1). The samples reached stable values within 30 min and did not exhibit any major change after that. In addition, the dissipation signal, Δ*D*, which is related to the viscoelasticity of the adsorbed layer, increased for all the samples, indicating that the adsorbed layer is coupled with the aqueous bulk. Nevertheless, as the dissipation is below 1 ppm, the Sauerbrey equation can be applied to evaluate the wet adsorbed mass to the quartz crystals, that is, the amount of chaperone coupled with water molecules, as reported in Figure 3. It is important to note that QCM-D measures the adsorbed amount in a more direct way than surface tension measurements.^48^ Thus, the lack of an induction time in QCM-D suggests that there was not a delay in adsorption *per se*.

**Figure 3:**
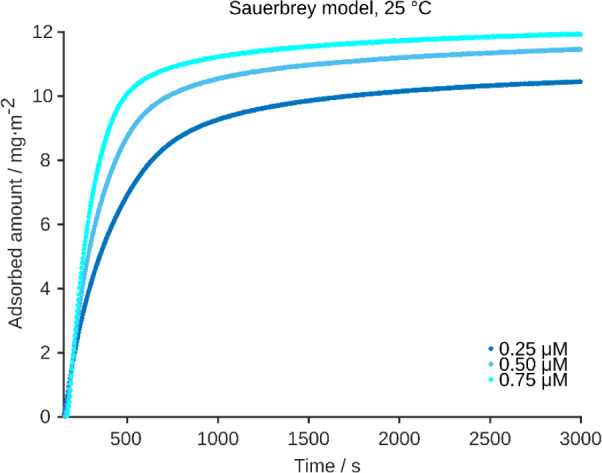
JB6 adsorption kinetics to silica surfaces based on QCM-D. While the overall adsorption time-scale is similar to the one from the dynamic surface tension measurements, no induction time was observed for 0.25 μM JB6. An induction time of hundreds of seconds would have been expected for this concentration in the surface tension measurements. The adsorbed mass increased somewhat with increasing JB6 concentration (from 0.25 to 0.75 μM) and started to plateau around about 9–11 mg·m^−2^. For the raw QCM-D frequency shifts and dissipation values, see Figure S1.

In the modeling of the protein adsorption, we associate the end of the induction time with a critical surface excess, Γ_0_. If we take the rate of adsorption to be diffusion limited and approximate the measured drop as a sphere, the amount of protein adsorbed after a specific time, *t*, is:^49,50^

1 $$\Gamma_{\mathrm{diff}}=\frac{c_{\mathrm{int}}\cdot R}{3}\cdot \left(1-\frac{6}{\pi^{2}}\cdot \overset{\infty }{\underset{z=1}{\sum }}\frac{\exp\left(\frac{-D\cdot z^{2}\cdot \pi^{2}\cdot t}{R^{2}}\right)}{z^{2}}\right)$$

Here, *c*_int_ is the initial, uniform bulk concentration of solute in the sphere, *D* is the diffusion coefficient, *R* is the radius of the droplet sphere (here 2.1 mm), and *z* is a summation variable without any immediate physical interpretation. Eq 1 is only expected to be applicable for the early stages of the adsorption, before the process becomes dominated by a free energy adsorption barrier (see the section on the theoretical model). The diffusion coefficient for JB6 has been estimated to be about 45 μm^2^/s at low concentrations.^9^ In reality, JB6 is present as a distribution of aggregates with a range of sizes. However, a diffusion-limited flux to a surface depends only weakly on the size of the clusters. It scales as *n* ^–1/6^ for a planar surface, where *n* is the number of monomers in a spherical cluster (*D* scales as *n* ^–1/3^ and the flux at a given time scales as *D* ^1/2^ .^37^ Therefore, we take the diffusion coefficient to be constant as a first approximation. By fitting the above relationship to the induction time as a function of bulk concentration we obtain a good agreement and an estimated critical surface coverage of about Γ_0_ = 24 × 10^3^ protein molecules per μm^2^ (1.1 mg/m^2^) at the end of the induction time (Figure 1b). This can be converted to an area per protein molecule of approximately 42 nm^2^, corresponding to a footprint with a radius of 3.6 nm. This is indeed similar to the size of a JB6 molecule (Figure 2b).

Due to the low concentrations of protein used in the measurements, it is important to consider the extent of depletion from the bulk of the drop.^51^ Eq 1 already incorporates such potential depletion of the bulk, since it is based on a finite volume (with a spherical geometry). Based on the estimated value of Γ_0_, the bulk concentration is expected to be decreased by about 0.05 μM at the end of the induction time for a drop of the size used in the measurements (50 mm^2^ area and 40 μL volume). Thus, the effect of depletion of the bulk is only expected to be substantial for the lowest concentrations used.

### Apparent Irreversibility of the Adsorption

Next, we consider the characteristics of the adsorbed protein layer. Small-sized surfactants generally adsorb reversibly to the air–water interface, such that the surface tension clearly stabilizes at an equilibrium value. However, already since the time of Irving Langmuir the adsorption of proteins to the air–water interface has been noted to be practically irreversible.^41,42^ This behavior is probably a main reason for the slow continued decrease of the surface tension with time for proteins. As thermodynamic equilibrium is not reached within the time of the measurement, protein molecules slowly continue to accumulate at the interface, causing an increased surface pressure and thus a decreased surface tension. We tested the reversibility of the adsorbed JB6 protein layer by using a coaxial capillary in the hanging drop tensiometer (Figure 4). First, an adsorbed layer of JB6 from a 0.44 μM bulk solution was allowed to form at the surface and reach the third, semistable, kinetic regime. Then, the bulk solution of the drop was exchanged with buffer. If the adsorption had been reversible, this would have resulted in an increase of the surface tension back toward the value of the buffer. However, in our measurement the surface tension remained unchanged, indicating that the protein was irreversibly adsorbed on the timescale of the experiment. On the contrary, exchanging the bulk with a higher JB6 concentration, 5.0 μM, allowed the surface tension to decrease further, even though the third kinetic regime had initially already been reached (Figure 4). Thus, while the adsorbed protein cannot be easily removed from the surface, the surface layer is not “locked” since additional protein molecules still can continue to adsorb from the bulk.

**Figure 4:**
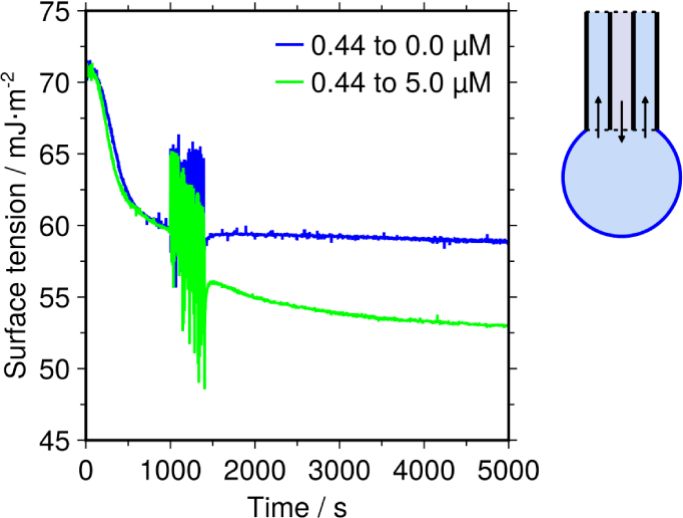
Apparent irreversibility of the adsorbed JB6 protein layer. A drop with 0.44 μM JB6 was formed using a coaxial capillary. After the adsorption had reached the semistable kinetic regime the bulk of the drop was exchanged to either pure buffer (blue) or 5.0 μM JB6 (green) during the time interval 1000–1400 s. Exchanging the bulk to pure buffer did not result in an increase of the surface tension, indicating irreversible adsorption of the surface film on the timescale of the experiments. Exchange to 5.0 μM JB6 resulted in a decrease of the surface tension. Thus, while adsorbed protein could not be removed, more protein could still be added.

The irreversible nature of the adsorption might be similar to that of long, nonprotein polymers. In that case, the binding between a single repeat unit and the interface can be relatively weak and reversible. Yet, the entire polymer chain can still be essentially irreversibly adsorbed due to the summed interactions of all repeat units with the interface.^54^ For the polymer to desorb, multiple repeat units need to be detached simultaneously.

### Elasticity of the Surface Layer

We characterized the mechanical nature of the adsorbed protein layer in terms of its elasticity. This was done by contracting or expanding the surface area of the drop through withdrawal or injection of liquid in its bulk. When reducing the size of a drop, the protein surface excess, Γ, was taken to be inversely proportional to the area, *A*, of the drop. If the adsorption had been readily reversible over the time-scale of the volume change, this relationship would not have been valid, since protein would desorb and reestablish equilibrium with the bulk solution. In the first contraction experiment, a drop with 0.44 μM JB6 was allowed to reach the third kinetic regime, in which the surface tension value had partially stabilized. Then the drop was contracted in several steps to reduce the surface area. This first resulted in an instantaneous decrease of the surface tension, followed by an upward, partial relaxation of the surface tension value (Figure 5a). This type of measurement was repeated for a drop with 5.0 μM JB6 (Figure 5b). With this setup we could only change the surface tension by decreasing the surface area. If the surface area was instead expanded, additional protein could easily adsorb to the surface and recover the initial surface tension value (Figure S3). This reinforced the results from the previous reversibility experiment where we showed that while adsorbed protein could not be removed, more protein could still be added. To circumvent this behavior in the elasticity measurements, we used the coaxial capillary and exchanged the bulk of the drop, so that there was only a shell of the protein layer at the surface with essentially pure buffer in the bulk. This allowed both contraction and expansion of the surface with Γ still considered inversely proportional to *A*. Thus, the elasticity of the surface layer could be probed also at surface tensions close to 72.0 mJ/m^2^, which is the surface tension value for the pure buffer (Figure 5c). It is noteworthy that the surface tension stayed almost constant over many thousands of seconds after the final change of area of the drop. This indicates that structural changes within the surface film are not the primary reason for the gradual decline in surface tension during the third kinetic regime.

**Figure 5:**
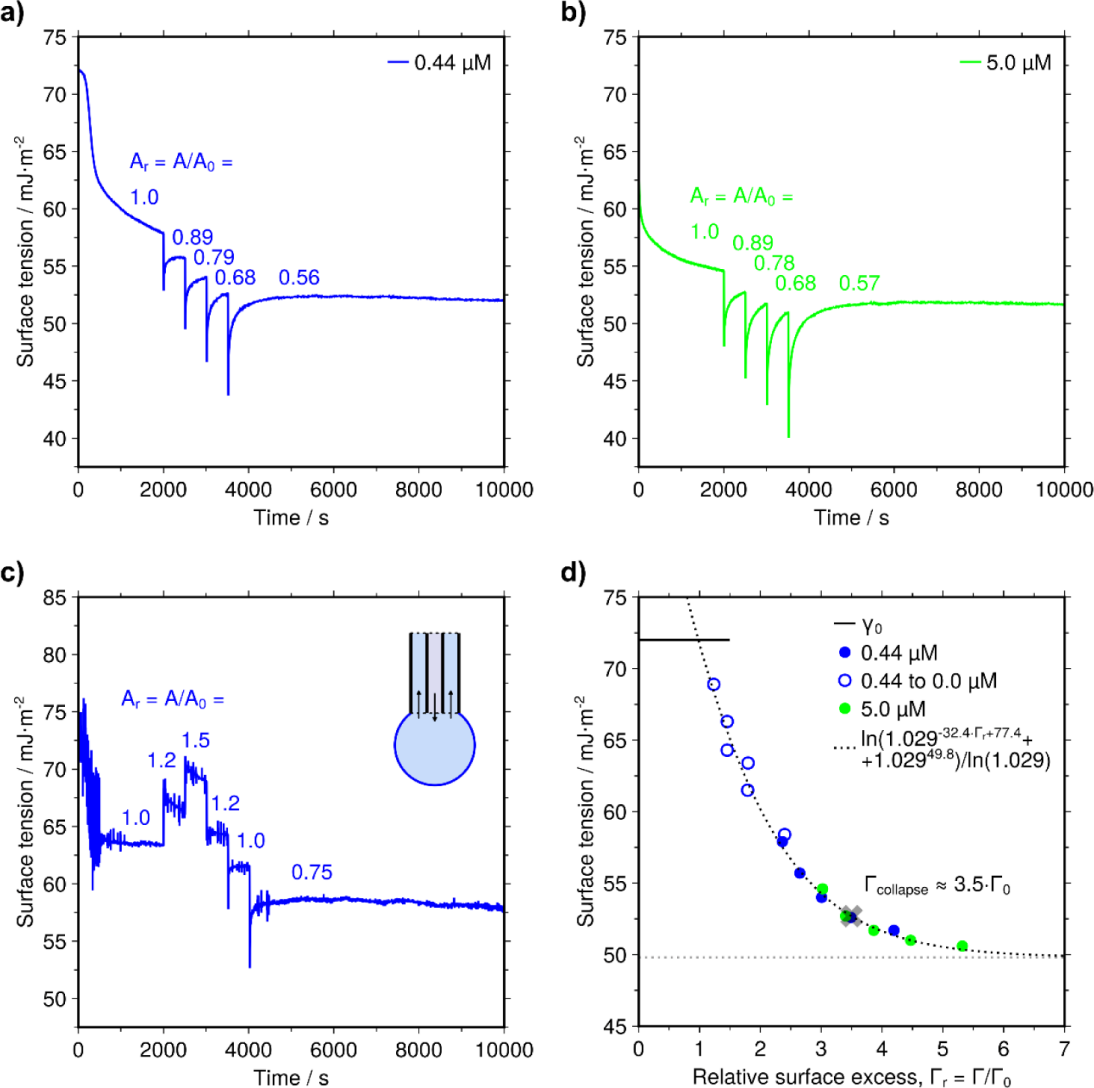
Elasticity of the adsorbed surface layer. **a)** Surface tension of a 0.44 μM JB6 drop with 4 steps of surface area contraction. **b)** Surface tension of a 5.0 μM JB6 drop with 4 steps of contraction. **c)** Expansion and contraction of a JB6 shell on buffer. The shell was formed by exchanging a 0.44 μM JB6 solution with buffer just after the formation of the drop. The numbers in **a–c** show the relative surface area of the drop, *A*_r_. **d)** The data from the measurements were combined into a model curve of the surface tension as a function of protein amount in the surface layer. For each measurement, Γ = α_*x*_·(1/*A*_r_), where α_*x*_ was a scaling factor for each set of points such that all points fell on a single curve (α_a_ = 2.36·Γ_0_, α_b_ = 3.02·Γ_0_, and α_c_ = 1.80·Γ_0_). Γ_0_ was 24 × 10^3^ μm^–2^, and Γ_collapse_ was 3.5·Γ_0_.

Combining the measurements above allowed for the construction of a single elasticity model curve, in which the surface tension was plotted as a function of the surface excess (Figure 5d). The estimated two-dimensional bulk elastic modulus was 15 mJ/m^2^ at the critical surface coverage, Γ_0_. It should be noted that the full mechanical behavior is likely more complex than what can be captured by these measurements.^33,55^ Nevertheless, this mechanical characterization serves as a first order description of the system. A surface layer is typically able to withstand a surface pressure only up to a point before it starts to collapse.^42,56,57^ We can see that the model curve gradually flattens out at a surface tension of about 50 mJ/m^2^, corresponding to a maximum surface pressure of Π = γ_0_ – γ = 22 mJ/m^2^. Furthermore, the maximum surface pressure might also correspond to the stage at which adsorption finally becomes notably reversible (through adsorbed multilayers or collapsed parts of the film being able to dissolve back into the bulk solution). A maximum surface pressure of around 20 mJ/m^2^ is in fact a typical value for proteins at the air–water interface.^34,35^ The overall shape of the model curve in Figure 5d is also similar to Π–Γ curves for several other proteins (for example lysozyme).^36^

Based on the induction time, we already estimated the area per protein molecule at the initial surface coverage (Γ_0_ = 24 × 10^3^ μm^–2^, 42 nm^2^, Figure 2b). With the additional information obtained from the mechanical measurements, the area per protein molecule at the point when the surface layer starts to reach its maximum surface pressure can also be estimated, yielding about Γ_collapse_ = 84 × 10^3^ protein molecules per μm^2^ (3.8 mg/m^2^). This corresponds to 12 nm^2^ per protein molecule and a footprint radius of 1.9 nm (Figure 2b). This is similar to the size of the folded *N*- and C-terminal domains of JB6. In the QCM-D measurements the adsorption started to plateau at roughly 10 mg/m^2^ for the three concentrations tested, as shown in Figure 3. The apparent difference between this value and the one extracted through the mechanical tests could be explained considering that QCM-D measures the mass of chaperone coupled with water, that is, wet mass. In addition, the interface in QCM-D is water–silica, therefore other phenomena may occur and the interaction between silica and the chaperone may be different, thus affecting the total amount adsorbed at the interface. In addition, the elasticity of the adsorbed layer can be qualitatively evaluated from the dissipation shift of the QCM-D curves. Overall, the system seems rigid (Δ*D* < 1 ppm) at all concentrations, pointing again to the different nature of the interface.

### Theoretical Model

The end of the induction period is taken as the time at which there is an initial complete monolayer coverage of protein at the air–water interface, formed by diffusion-limited adsorption. The subsequent decrease of the surface tension indicates the continued adsorption of protein from the bulk, limited by an adsorption barrier due to lateral compression of the surface layer. The amount of protein for each surface tension value, or surface pressure, can be estimated based on the elasticity curve (Figure 5d). Within the model, we take the adsorption of JB6 to the air–water interface to be fully irreversible. To make space for incoming material, cavitation against the surface pressure of the surface layer is required, with a free energy change Δ*G*_cav_. Furthermore, a compression of the incoming material, corresponding to a free energy change Δ*G*_comp_, to equalize with the surface pressure of the surface layer is also necessary (Figure 6a). These processes entail a surface pressure dependent activation free energy barrier for adsorption. At each protein concentration in the bulk, the protein molecules have a certain chemical potential (larger for higher concentrations). When the chemical potential of the protein in the bulk is high relative to the top of the activation barrier for adsorption, addition of more material to the surface layer is expected to be fast. When the chemical potential in the bulk is low relative to the top of the barrier, adsorption is slow. Based on this framework, we constructed a mathematical model of the system (Section S2). For each surface excess and bulk chemical potential of the protein near the surface, we obtain a rate of adsorption, expressed as:

**Figure 6:**
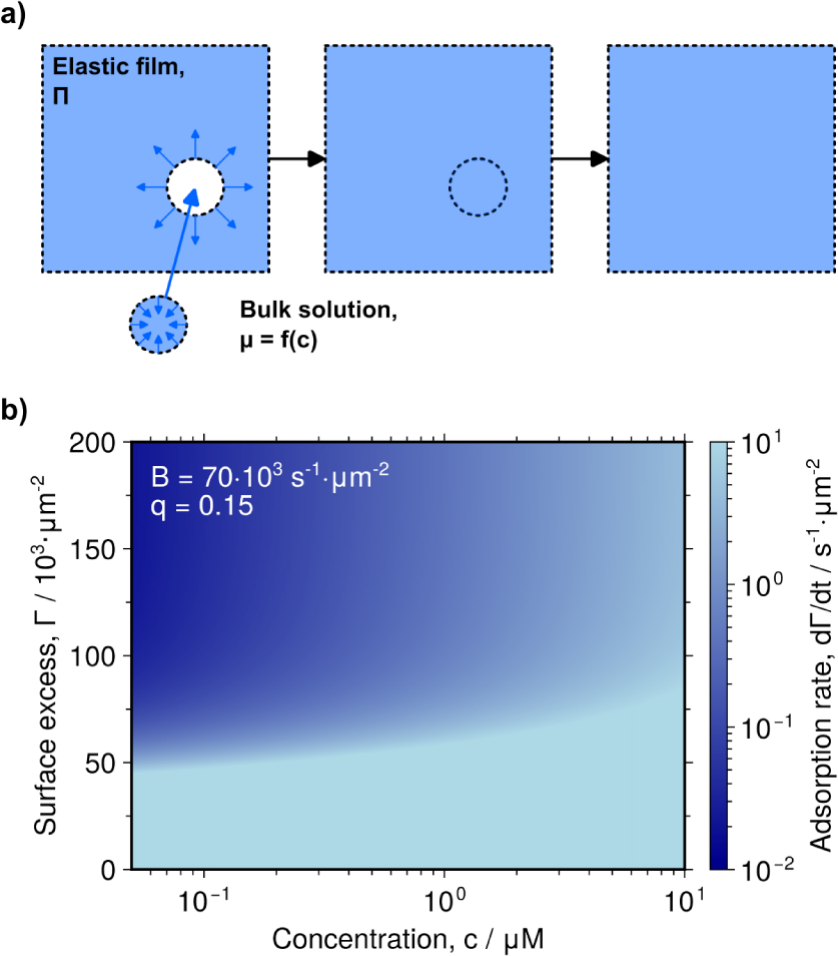
Framework for the proposed model. **a)** Schematic illustration of incorporation of additional material from the bulk to the surface layer. To add the new material, a cavity needs to be formed, and the incoming material is compressed by the surface pressure of the surface layer. The bulk solution is characterized by a concentration, *c*, and a corresponding chemical potential, μ. Once the free energy barrier for adsorption has been overcome, the new material is irreversibly incorporated into the surface layer. **b)** The adsorption rate as a function of bulk concentration and surface excess fitted to the experimental data for JB6, without considering the contribution from protein clustering.

2 $$\frac{d\Gamma }{dt}=B\cdot \exp\left(-\frac{q\cdot \left(\Delta G_{\mathrm{comp}}+\Delta G_{\mathrm{cav}}\right)}{k_{B}T}\right)\cdot \exp\left(\frac{\mu_{P_{\mathrm{bulk}}}-\mu_{P_{\mathrm{bulk}}}^{◦}}{k_{B}T}\right)$$

where *B* and *q* are fitting parameters (*B* = 70 × 10^3^ s^–1^·μm^–2^, *q* = 0.15, Figure 6b). If we approximate the bulk protein solution as ideal-dilute, we can express the bulk chemical potential as $\mu_{P_{\mathrm{bulk}}}=\mu_{P_{\mathrm{bulk}}}^{◦}+k_{B}T\cdot \ln c$ , with *c* being the total bulk protein concentration in monomer units next to the surface. We then combine these equations with diffusion to the surface of a sphere.

3 $$\frac{d\Gamma }{dt}=-D\cdot {\frac{\partial c}{\partial r}|}_{r=R}$$

4 $$\frac{\partial c}{\partial t}=D\cdot \left(\frac{2}{r}\cdot \frac{\partial c}{\partial r}+\frac{\partial^{2}c}{\partial r^{2}}\right)$$

where *D* is the diffusion coefficient, *r* is the radial distance from the center within the sphere, and *R* is the radius of the whole droplet sphere (2.1 mm, Figure 1). Simultaneously satisfying all equations yields the adsorbed amount of protein as a function of time. By converting the adsorbed amount to a surface tension in accordance with the relationship in Figure 5d, surface tension curves are obtained. Our approach is similar to that of MacRitchie and Alexander,^34,37–40^ which in turn was based on Ward and Tordai.^58^ They also used the concept of an adsorption barrier due to the surface pressure of the adsorbed layer. However, our model differs in that we also include a compression step of the incoming material in the adsorption barrier, do not consider the cavitation area to be constant, and incorporate a nonlinear relationship between surface pressure and surface excess. The curves obtained through the adsorption model capture most features of the experimental result (Figure 7a). However, we can only partially capture the plateauing of the surface tension as the protein concentration is increased. As mentioned in the introduction, JB6 is known to form micelle-like clusters in solution. This is expected to affect the adsorption behavior, and may contribute to the plateauing with increasing concentration.

**Figure 7:**
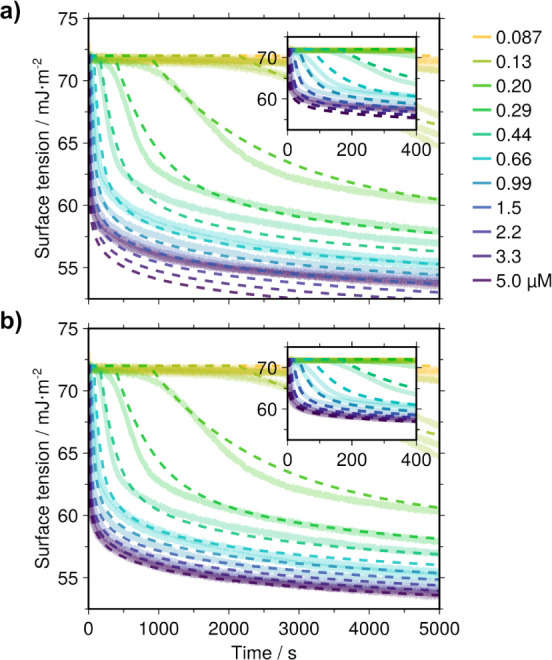
Numerically calculated dynamic surface tension curves based on the adsorption model. **a)** Calculation without protein clustering, with *B* = 70 × 10^3^ s^–1^·μm^–2^ and *q* = 0.15 (same as in Figure 6b). **b)** Calculation with clustering incorporated through the isodesmic association model with *K* = 5 × 10^–3^ μm^3^ (other parameters being the same as in **a**). The calculation from the model (dashed lines) is plotted on top of the experimental data (faint solid lines). The model can semiquantitatively reproduce the main features of the experimental curves.

The size distribution of the JB6 clusters is broad but not perfectly known.^9,11^ For such a situation an isodesmic association model is a reasonable first approximation (Section S3).^59^ In fact, it has been suggested to describe the association behavior of another chaperone protein (HSC70).^60^ The isodesmic association model is based on a single parameter, *K*, which is the equilibrium constant for addition of a monomer to a cluster of any size. Thus, this single parameter is a measure of the overall clustering propensity of the solute (higher *K* means higher clustering propensity). Each cluster of aggregation number *n* can act as an adsorption species contributing with *n* protein monomers to the surface layer. Since a cluster is a different and larger species than a monomer, the free energy barrier for adsorption is also expected to be different. The adsorption barrier can potentially vary in a complex way with the size of a cluster. It is known that JB6 solutions start to have a substantial number of clusters at around 0.1 μM under the same conditions as used in this study.^9^ This corresponds to a clustering propensity of roughly *K* = 3 μM^–1^ (or 5 × 10^–3^ μm^3^) in the isodesmic model. By using this as an input in our adsorption model, we find that the adsorption barrier must scale extremely weakly with increasing cluster size, for example as $\Delta G^{‡}\propto n^{1/20}$ . We thus model the adsorption kinetics with the equations (Section S2 and Figure 7b).

5 $$\frac{d\Gamma }{dt}=\overset{\infty }{\underset{n=1}{\sum }}\left(n\cdot B\cdot \exp\left(-\frac{n^{1/20}\cdot q\cdot \left(\Delta G_{\mathrm{comp}}+\Delta G_{\mathrm{cav}}\right)}{k_{B}T}\right)\cdot \exp\left(\frac{\mu_{n}-\mu_{n}^{◦}}{k_{B}T}\right)\right)$$

6 $$\frac{d\Gamma }{dt}=\overset{\infty }{\underset{n=1}{\sum }}\left(-n\cdot D\cdot {\frac{\partial c_{n}}{\partial r}|}_{r=R}\right)$$

7 $$\frac{\partial c_{n}}{\partial t}=D\cdot \left(\frac{2}{r}\cdot \frac{\partial c_{n}}{\partial r}+\frac{\partial^{2}c_{n}}{\partial r^{2}}\right)$$

Here, *c*_*n*_ is the concentration of clusters with *n* monomers, and we set $\mu_{n}=\mu_{n}^{◦}+k_{B}T\cdot \ln c_{n}$ . We do not consider transformations of clusters of different sizes into each other when the concentration changes, because the equilibration of cluster sizes of JB6 is known to be slow in the bulk at room temperature.^9^ The reason for the very weak scaling of the adsorption barrier with cluster size (scaling exponent 0.05) could be that the JB6 clusters lose one monomer at a time at the interface, rather than being incorporated into the interfacial layer in a single reaction step. Interestingly, in the modeling approach by MacRitchie and Alexander, the adsorption barrier was found to be almost constant for proteins irrespective of size.^34,38^ This was interpreted as only a segment of each protein, of similar size for all proteins, needing to reach into the surface layer in the transition state for adsorption to occur. However, within the scope of the present study, these mechanisms cannot be fully determined, and we leave it open for future investigations.

Both the experimental and modeled surface tension can be plotted as a function of scaled time by normalizing with the induction time for each curve (Figure S4).^33,61^ This makes it easier to identify the potential limitations of the model. At the beginning of the adsorption process, the behavior is dominated by diffusion. As the surface layer builds up there is a gradual transition to dominance by a free energy barrier. This eventually causes a reversal of the concentration dependence of adsorption relative to the diffusion-limited case. As can be seen in Figure S4, such a change in the concentration dependence happens already in the second kinetic regime of the experimental data, whereas it occurs later for the model curves. This might be because the model only takes the surface pressure into account, whereas the actual mechanical nature of the surface layer is more complex.^33,55^ For example, the viscous properties of the surface layer likely play a role when the adsorption rate is high. Indeed, for concentrations above approximately 0.5 μM JB6 the timescales of the rapid drop in surface tension are comparable to the mechanical relaxation time of the surface layer (see the peaks in Figures 5 and S3).

There are striking similarities between the adsorption behavior of proteins and nanoparticles at fluid interfaces.^33,61,62^ Both types of systems can exhibit effectively irreversible adsorption with a rate that is at first diffusion-limited and then the process slows down with time, due to a buildup of mechanical resistance to further adsorption from the bulk. Nelson et al.^61^ describe their nanoparticle system using a similar conceptual framework as in our model and mention the need for rearrangement within the surface layer to make space for more particles. Schwenke et al.^62^ arrived at similar conclusions based on computer simulations. Similarities in the adsorption behavior of proteins and nanoparticles are highly interesting, because such similarities point toward the relative insignificance of internal degrees of freedom for the adsorption behavior.

## Conclusions

JB6 is surface active and adsorbs to the air–water interface. The protein forms an effectively irreversibly adsorbed and elastic surface layer. The kinetics of adsorption exhibits three regimes. The first regime is the induction time when the surface tension stays close to the value of the buffer. As a first approximation, it can be modeled as diffusion-limited adsorption combined with a two-dimensional phase separation. In the second regime there is a relatively rapid decrease of the surface tension, and it can be understood as the bulk chemical potential of the protein being high relative to the top of a surface pressure dependent free energy barrier for adsorption. The third regime corresponds to a slow continued decrease of the surface tension. It can be seen as the bulk chemical potential being low relative to the top of the adsorption barrier. At high concentrations the surface tension converges on a minimum value. Both the collapse of the surface layer and the clustering of JB6 contribute to this limiting value in the surface tension. The surface area per molecule at the end of the induction time is similar to the size expected for a JB6 molecule in the bulk, whereas the area when the surface layer starts to collapse is similar to the size of the folded domains within the protein.

This study represents a first step in the interface-focused approach to unveil the mechanisms of chaperones. Future steps along this line could be inclusion of a client molecule, such as an amyloidogenic peptide, in the experiments. Overall, the adsorption of JB6 to the air–water interface appeared similar to that of other proteins. Thus, the model we developed to understand the results could also have wider applicability in other contexts where protein adsorption plays a role, such as in material and pharmaceutical science.

## Supplementary Material



## Data Availability

The data are available at https://github.com/saralinse/Published_Data/tree/Langmuir_2025_JB6_aiw_adsorption.

## Notes

QCM-D data, Brewster angle microscopy images, additional surface tension measurements related to mechanical properties, further analysis of dynamic surface tension curves, and details about the theoretical model (PDF)
