# Supplementary material for: Air–Water Interfacial Adsorption of the Chaperone Protein DNAJB6b
Source: Langmuir. 2025 Jul 16;41(29):19146–55. doi: 10.1021/acs.langmuir.5c01237 (PMC12312157; doi:10.1021/acs.langmuir.5c01237)
Supplement: Supplementary file 1 [file la5c01237_si_001.pdf]

# Supplementary information

# Air-water interfacial adsorption of the chaperone protein DNAJB6b

*Jon Pallbo[1*], Marco Fornasier[1], Sara Linse[2], Ulf Olsson[1]*

[1]Physical Chemistry, Lund University, P.O. Box 124, 221 00 Lund, Sweden

[2]Biochemistry and Structural Biology, Lund University, P.O. Box 124, 221 00 Lund, Sweden

[*]jon.pallbo_arvidsson@fkem1.lu.se

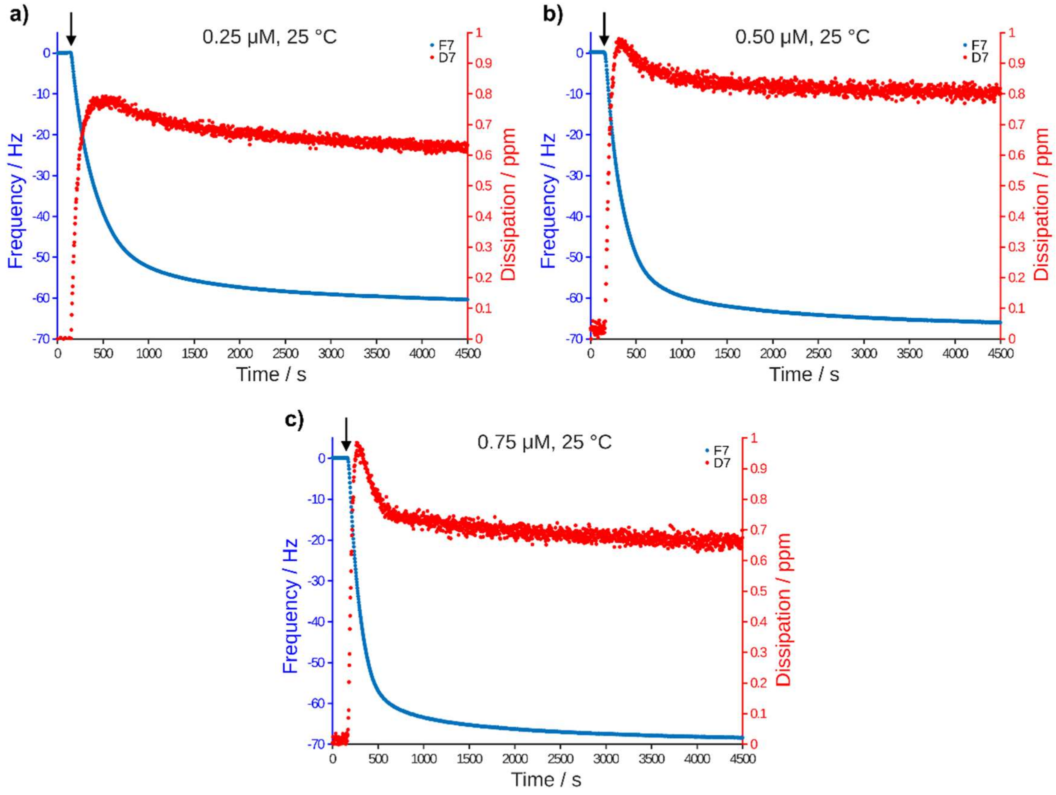


**Figure S1.** QCM-D data regarding the adsorption of JB6 to plain silica (hydrophilic) at 0.25 (**a**), 0.50 (**b**) and 0.75 µM (**c**). The plot shows the trend of the frequency (teal dots) and dissipation (red dots) for the representative 7$^{th}$ overtone of the system, while the arrow represents the injection of the chaperon in the cells at a flow rate of 150 mL/min after a baseline obtained under buffer flow. The measurements were performed at 25 °C.

## Section S1. Brewster angle microscopy

DNAJB6b (JB6) was expressed and isolated in the same way as for the other experiments, except that a protease inhibitor cocktail (cOmplete EDTA-free, Roche Diagnostics GmbH, Germany) was included in all purification steps apart from the final one. A frozen aliquot of JB6 (65 μM in buffer) was thawed overnight and a small drop of about 1 μL was touched onto the surface of a buffer solution with a surface area of about 36 $cm^2$. This resulted in partial coverage of the surface with a JB6 film. The surface was observed through Brewster angle microscopy using an Optrel Multiskop (Optrel GBR, Germany). The structures on the surface moved around due to convection streams, and images were captured as the edge of the JB6 film floated across the field of view (**Figure S2**).

The purpose of the experiment was to determine whether JB6 spreads on the surface as a two-dimensional gas or as a two-phase system with a condensed phase (see **Figure 2a** in the main document). The observation of an edge of the JB6 surface film in the field of view indicates the presence of two-phases (condensed and gaseous). The direction of movement further supports this conclusion, because if the edge had been due to the spreading out of a gaseous phase, the movement would have been in the opposite direction.

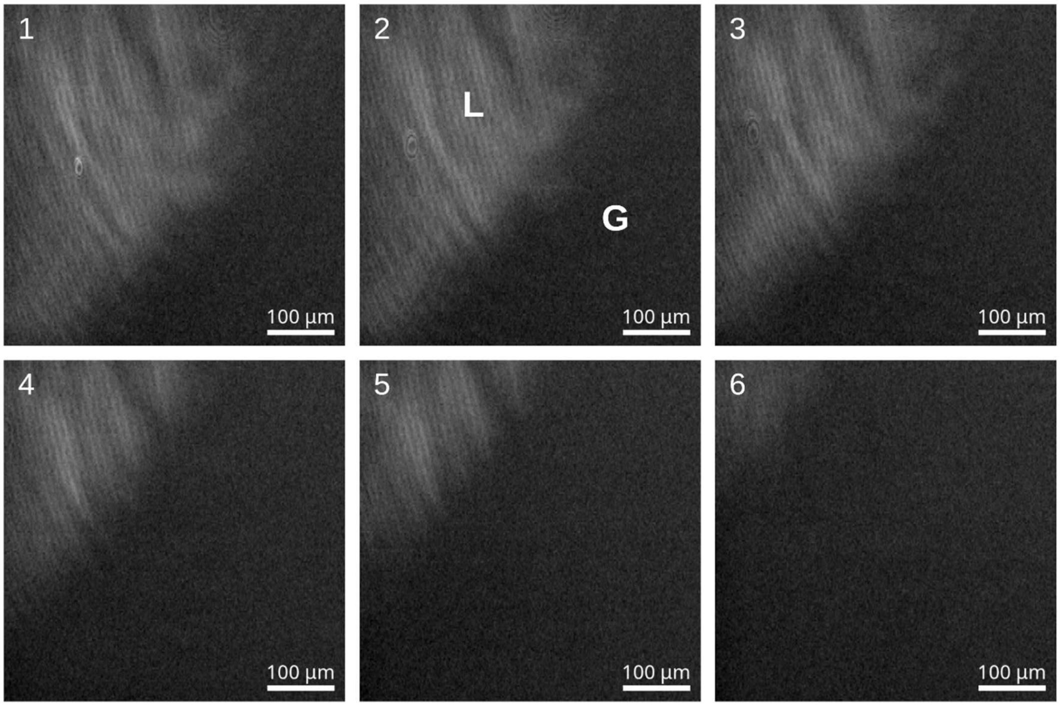


**Figure S2.** Brewster angle microscopy evidence of two-dimensional phase separation of JB6. A small amount of JB6 was spread onto the surface of a buffer solution, resulting in partial coverage of the surface with a condensed film (L) surrounded by a two-dimensional gas phase indistinguishable from the buffer alone (G). A chronological sequence of images (1 to 6) was then captured as the edge of the surface film floated across the field of view. The total duration of the sequence was about 6 seconds.

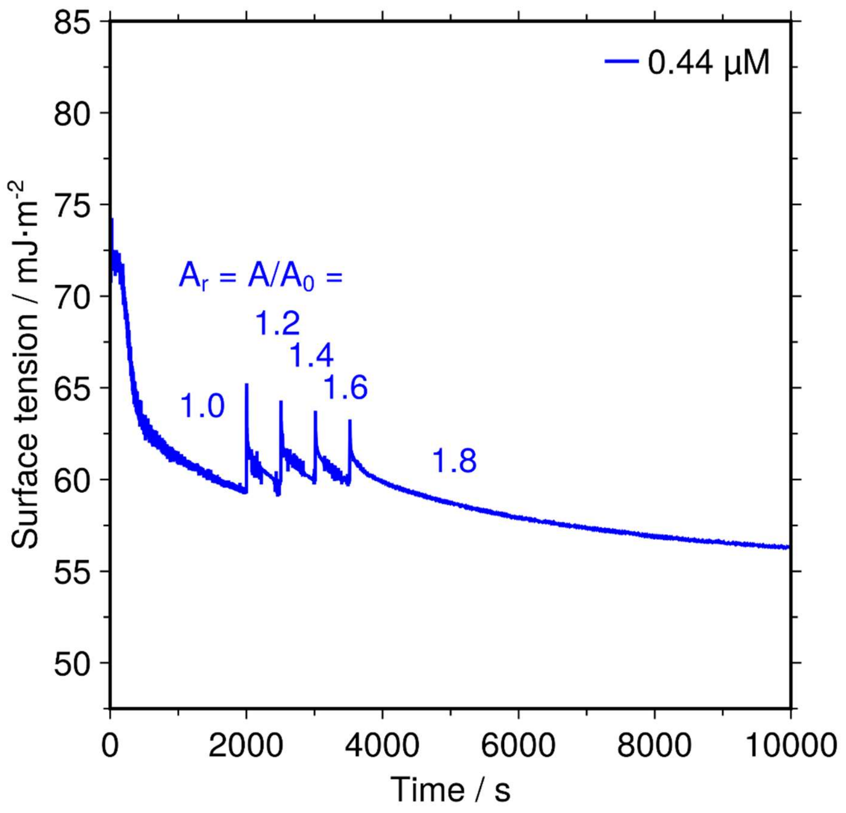


**Figure S3.** Surface tension of a drop of 0.44 μM JB6 with 4 steps of surface area expansion without using the coaxial capillary. The surface tension recovered after each step, indicating that protein could easily adsorb from the bulk after each surface expansion. $A_r$ is relative surface area (the area of the drop divided by the initial area of the drop).

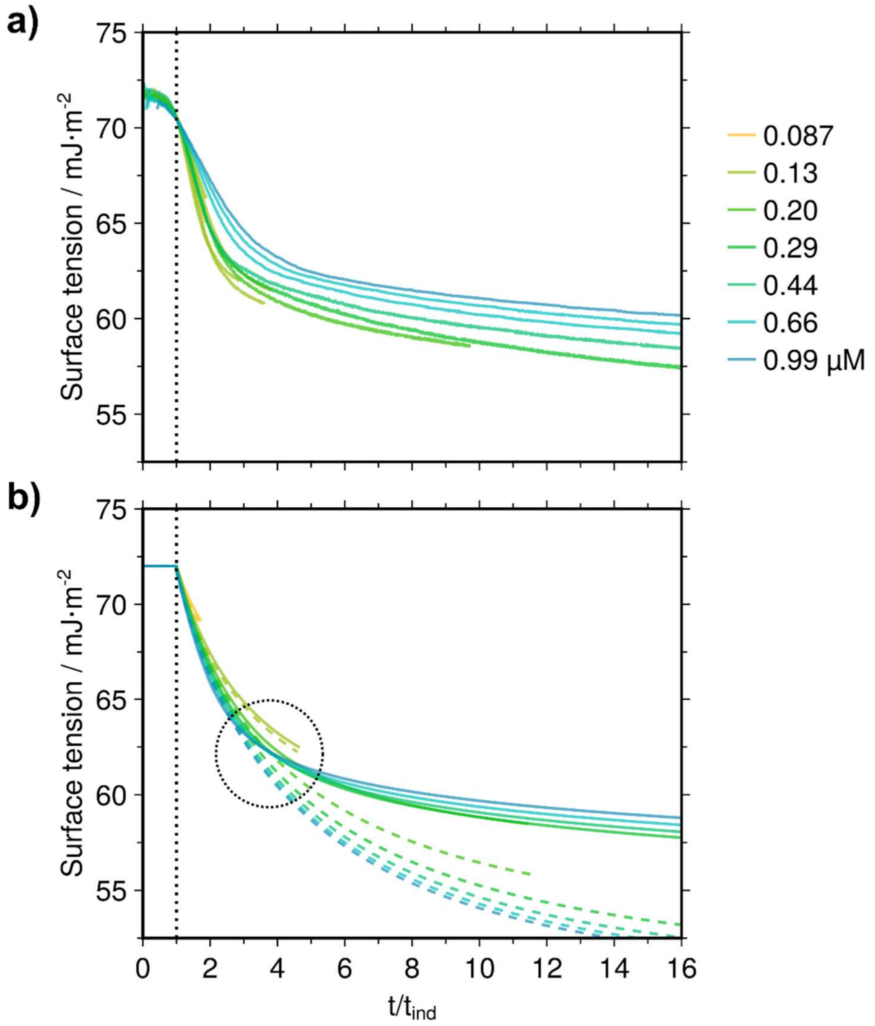


**Figure S4.** Surface tension as a function of scaled time. **a)** Experimental data with the time, $t$, normalized by the induction time, $t_{ind}$, for each curve (for the samples with a measurable induction time). **b)** Model curves from **Figure 7b** in the manuscript (solid lines) plotted in the same way as the experimental data, and curves for diffusion limited adsorption (dashed lines). As the adsorption progresses, there is a transition to barrier-dominated kinetics and a reversal of the order of the curves relative to the diffusion limited case (circle). This happens earlier for the experimental data than for the model curves, which might be because the model does not fully capture the complexity of the mechanical properties of the adsorbed surface layer. In the case of an infinite system with a planar surface, the diffusion-limited curves would collapse onto a single master curve by this normalization.

**Section S2. Derivation of the adsorption rate expression**

The adsorption of the protein to the surface can be represented by the process

$$P_{bulk} \rightleftharpoons P^{\ddagger}_{surf} \rightarrow P_{surf} \qquad \text{(Eq. S1)}$$

where $P_{bulk}$ is the protein in the bulk solution, $P^{\ddagger}_{surf}$ is the protein in the transition state for adsorption to the surface, and $P_{surf}$ is the protein incorporated into the surface layer. We express the free energy barrier, $\Delta G^{\ddagger}$, associated with the adsorption as

$$\Delta G^{\ddagger} = \Delta G_0 + q \cdot \left(\Delta G_{comp} + \Delta G_{cav}\right) \qquad \text{(Eq. S2)}$$

$$\Delta G_{comp} = -\int_{a=\frac{1}{\Gamma_0}}^{\frac{1}{\Gamma}} \Pi \cdot da \qquad \text{(Eq. S3)}$$

$$\Delta G_{cav} = \frac{\Pi}{\Gamma} \qquad \text{(Eq. S4)}$$

where $\Delta G_0$ is the free energy barrier at zero surface pressure and $q$ is a fitting parameter (**Figure S5**). $\Delta G_{comp}$ is the free energy of compressing incoming material (from the area per molecule at zero surface pressure to the area per molecule at the surface pressure of the surface layer) and $\Delta G_{cav}$ is the free energy to make a cavity in the surface layer for the incoming material. Γ and Π are the protein surface excess and surface pressure, respectively. We then express the rate of adsorption as an activated process in the form

$$\frac{d\Gamma}{dt} = \beta \cdot \exp\left(-\frac{\Delta G^{\ddagger}}{k_B T}\right) \cdot \exp\left(\frac{\mu_{P_{bulk}} - \mu^{\circ}_{P_{bulk}}}{k_B T}\right) = \qquad \text{(Eq. S5)}$$

$$= \beta \cdot \exp\left(-\frac{\Delta G_0 + q \cdot \left(\Delta G_{comp} + \Delta G_{cav}\right)}{k_B T}\right) \cdot \exp\left(\frac{\mu_{P_{bulk}} - \mu^{\circ}_{P_{bulk}}}{k_B T}\right) =$$

$$= B \cdot \exp\left(-\frac{q \cdot \left(\Delta G_{comp} + \Delta G_{cav}\right)}{k_B T}\right) \cdot \exp\left(\frac{\mu_{P_{bulk}} - \mu^{\circ}_{P_{bulk}}}{k_B T}\right)$$

where $\beta$ and $B$ are constants ($B$ is used as a fitting parameter).

From the experimental results we found that the adsorption at zero surface pressure (during the induction time) agrees with diffusion-limited adsorption. This suggests that $\Delta G^{\ddagger}$ is dominated by the term $q \cdot (\Delta G_{comp} + \Delta G_{cav})$, which is the reason $\Delta G_0$ was not included when modelling the adsorption with clustering. If $\Delta G_0$ had been included, parameter $B$ would have different values in the no-clustering and clustering case (**Eq. 2** and **Eq. 5** in the main text).

## Section S3. Isodesmic association model

In a stepwise association model, the association process is represented by

$$M_1 + M_{n-1} \rightleftharpoons M_n \qquad \text{(Eq. S6)}$$

where $M_n$ is a cluster with $n$ protein monomers. Each step is associated with an equilibrium constant, $K_i$, which in the isodesmic model is the same for all steps ($K_2 = K_3 = K_4 = \ldots = K$) The concentration of $n$-sized clusters, $c_n$, can be obtained through

$$c_n = \left(\prod_{i=2}^{n} K_i\right) \cdot {c_1}^n = K^{n-1} \cdot {c_1}^n \qquad \text{(Eq. S7)}$$

$$c_1 = \frac{1 + 2 \cdot K \cdot c_{tot} - \sqrt{1 + 4 \cdot K \cdot c_{tot}}}{2 \cdot K^2 \cdot c_{tot}} \qquad \text{(Eq. S8)}$$

where $c_{tot}$ is the total concentration of protein molecules given by

$$c_{tot} = \sum_{n=1}^{\infty}(n \cdot c_n) = \sum_{n=1}^{\infty}(n \cdot K^{n-1} \cdot {c_1}^n) = \frac{c_1}{(K \cdot c_1 - 1)^2}. \qquad \text{(Eq. S9)}$$

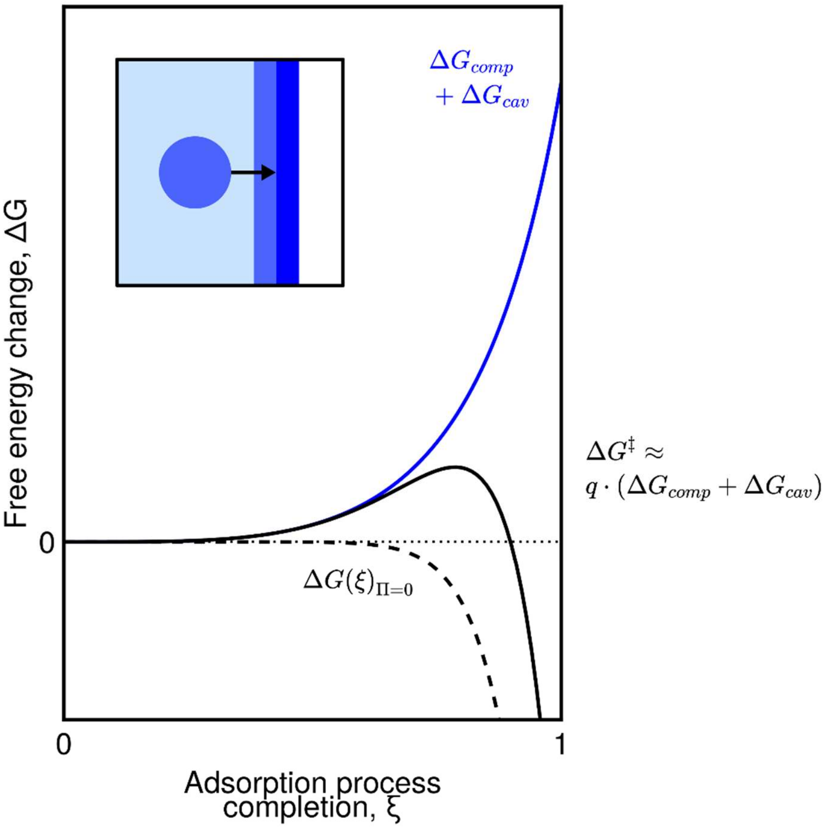


**Figure S5.** Schematic illustration of the adsorption barrier and the physical origin of the *q* parameter used in the model. As a particle from the bulk adsorbs at the interface, it experiences both favorable and unfavorable interactions. The black dashed curve represents the interaction with the interface at zero surface pressure ($\Pi = 0$), which is dominated by strong short-ranged attraction. The blue solid curve represents the free energy cost of compression of the adsorbing material ($\Delta G_{comp}$) and of making a cavity in the surface layer ($\Delta G_{cav}$). The black solid curve is the sum of the other curves and represents the interaction with the interface at non-zero surface pressure. It exhibits a free energy barrier ($\Delta G^{\ddagger}$). The *q* parameter in the expression for the free energy barrier arises because of the overlap with favorable interactions.
